# Comparison of Visceral Leishmaniasis Diagnostic Antigens in African and Asian *Leishmania donovani* Reveals Extensive Diversity and Region-specific Polymorphisms

**DOI:** 10.1371/journal.pntd.0002057

**Published:** 2013-02-28

**Authors:** Tapan Bhattacharyya, Marleen Boelaert, Michael A. Miles

**Affiliations:** 1 Faculty of Infectious and Tropical Diseases, London School of Hygiene and Tropical Medicine, London, United Kingdom; 2 Department of Public Health, Institute of Tropical Medicine, Antwerp, Belgium; Institute of Tropical Medicine, Belgium

## Abstract

**Background:**

Visceral leishmaniasis (VL), caused by infection with *Leishmania donovani* complex, remains a major public health problem in endemic regions of South Asia, East Africa, and Brazil. If untreated, symptomatic VL is usually fatal. Rapid field diagnosis relies principally on demonstration of anti-*Leishmania* antibodies in clinically suspect cases. The rK39 immunochromatographic rapid diagnostic test (RDT) is based on rK39, encoded by a fragment of a kinesin-related gene derived from a Brazilian *L. chagasi*, now recognised as *L. infantum*, originating from Europe. Despite its reliability in South Asia, the rK39 test is reported to have lower sensitivity in East Africa. A reason for this differential response may reside in the molecular diversity of the rK39 homologous sequences among East African *L. donovani* strains.

**Methodology/Principal Findings:**

Coding sequences of rK39 homologues from East African *L. donovani* strains were amplified from genomic DNA, analysed for diversity from the rK39 sequence, and compared to South Asian sequences. East African sequences were revealed to display significant diversity from rK39. Most coding changes in the 5′ half of repeats were non-conservative, with multiple substitutions involving charge changes, whereas amino acid substitutions in the 3′ half of repeats were conservative. Specific polymorphisms were found between South Asian and East African strains. Diversity of HASPB1 and HASPB2 gene repeat sequences, used to flank sequences of a kinesin homologue in the synthetic antigen rK28 designed to reduce variable RDT performance, was also investigated. Non-canonical combination repeat arrangements were revealed for HASPB1 and HASPB2 gene products in strains producing unpredicted size amplicons.

**Conclusions/Significance:**

We demonstrate that there is extensive kinesin genetic diversity among strains in East Africa and between East Africa and South Asia, with ample scope for influencing performance of rK39 diagnostic assays. We also show the importance of targeted comparative genomics in guiding optimisation of recombinant/synthetic diagnostic antigens.

## Author Summary

Visceral leishmaniasis (VL) is caused by infection with parasites of the *Leishmania donovani* complex, spread by the bite of blood-sucking sandflies, especially in South Asia, East Africa, and Brazil. If untreated, symptomatic VL leads to systemic pathologies and is usually fatal. Up to approximately 400,000 new cases are estimated to occur annually, and regional epidemics have been devastatingly severe. Diagnosis of clinically suspect cases in the field, and thus appropriate treatment, relies principally on a rapid diagnostic test (RDT) based on detection of antibodies against a *Leishmania* antigen known as rK39. Although this test is reliable in South Asia, it has shown less success in East Africa. One reason may reside in diversity of the rK39 homologue among East African *L. donovani*, with consequent limitations in the binding of diagnostic antibodies. Here, we investigate the sequences of rK39 homologues in a panel of East African *L. donovani* strains, and show that there is significant diversity compared to rK39 and to South Asian sequences. Additionally, we examine diversity in another diagnostic antigen known as HASPB1/2. Our results indicate that an improved RDT may need to encompass East-African-specific antigen diversity to provide high performance field diagnosis for this region.

## Introduction

Visceral leishmaniasis (VL) remains a major public health concern in many parts of the tropical world, with the great majority of the estimated 200,000 to 400,000 annual new cases found in South Asia, East Africa, and Brazil [1], [2]. VL is caused by kinetoplastid protozoa of the *Leishmania donovani* complex. These are: *L. donovani* in South Asia and East Africa; *L. infantum*, mainly in Europe, the wider Mediterranean region, and in Latin America, where it was historically also known as *L. chagasi* but is now demonstrated to be synonymous with *L. infantum* originating from Europe [3].

*Leishmania* promastigotes, transmitted during bloodmeal feeding by female sandflies (*Phlebotomus* and *Lutzomyia* spp., in the Old and New World respectively), are internalised by local dermal macrophages and dendritic cells. Within these host cells, flagella are lost, and transformation into proliferative amastigote forms is followed by cell lysis, re-invasion of other cells, and parasite dissemination by the lymphatic and vascular systems, which can lead to infiltration of bone marrow and hepatosplenomegaly. In symptomatic cases, VL is fatal if untreated [4].

Parasitological diagnosis, by demonstration of amastigotes in spleen aspirates, approximates to a gold standard for VL diagnosis but is applied cautiously due to associated risk. Serological (anti-*Leishmania* antibody) tests include enzyme-linked immunosorbent assay (ELISA), indirect fluorescent antibody test (IFAT), and direct agglutination test (DAT) [5]. However, after drug treatment and cure current serological tests may still give positive results and therefore cannot readily diagnose relapse. Furthermore, such tests can also detect anti-leishmanial antibodies in asymptomatic individuals living in endemic areas, but with no VL history or subsequent progression to VL [6].

Burns et al [7] identified a kinesin-related gene product, LcKin, as a candidate diagnostic antigen by screening a Brazilian *L. infantum* (*L. chagasi*) genomic library with serum of an *L. donovani* patient. A part of the coding sequence, comprising a 46aa region followed by 6.5×39aa repeats, forms the recombinant diagnostic protein rK39. In recent multicentre evaluations, the use of the rK39 in a lateral-flow immunochromatographic, rapid test format reported less success in East Africa than in the Indian subcontinent for point-of-care diagnosis of VL [8], [9]. Underlying explanatory factors may reside in molecular divergence between East African *L. donovani* kinesin gene homologues and the Brazilian *L. infantum* (*L. chagasi*)-derived rK39 sequence, and/or may be due to differential immunocompetence and antibody levels produced among African and Asian human populations.

Studies on South Asian *L. donovani* strains using PCR primers based on *LcKin* have identified rK39 homologous sequences [10]–[12]. Gerald et al [13] reported the first East African (Sudanese) *L. donovani* kinesin homologue, LdK39. The first two of the 39-aa repeats of LdK39, flanked by sequences of the *L. donovani* antigens HASPB1 and HASPB2 [14], comprise rK28, a novel recombinant protein for diagnosis of VL, designed to be an improvement over rK39 [15]–[16]. HASPB proteins are expressed on the surface of infective promastigote and amastigote *Leishmania* life cycle stages [14]. The first 3×14aa repeats of HASPB1 are incorporated into rK28, along with the complete ORF of HASPB2, which includes three imperfect consecutive repeats, 2×14aa and 1×13aa. HASPB1 and HASPB2 correspond with K26 and K9, respectively [17], which were originally identified in *L. infantum* (*L. chagasi*) at the same time as the HASPBs, but were given different nomenclature. A recent comparison of rK26 and rK9 showed significantly lower diagnostic efficacy than rK28 [15].

Here we investigate whether molecular divergence in rK39 kinesin sequence homologues of East African *L. donovani* may contribute to lower rK39 diagnostic test success rates in East Africa. We analyse the rK39 homologues in a panel of East African *L. donovani* strains and compare their diversity against published South Asian sequences. In addition, we compare sequence diversity of HASPB1 and HASPB2 in East African and South Asian strains.

## Methods

### *L. donovani* strains

Table 1 lists the East African *L. donovani* strains used in this study. Strains were selected to represent genetic groups within the *L. donovani* complex in East Africa (Baleela et al, unpublished data; identified by multilocus sequence typing (MLST) and microsatellite analysis (MLMT)). Strains were cultured in αMEM medium supplemented by foetal calf serum (Sigma, UK), and genomic DNA was extracted from uncloned cultures using Gentra Puregene Tissue Core Kit A (Qiagen, UK). Uncloned cultures were specifically used here because the intention was to capture the diversity present within natural *L. donovani* populations. Table 2 lists the GenBank sequences derived in other studies [7], [10]–[14], [18]–[19] and used here for comparisons.

**Table 1:** East African *L. donovani* strains for which sequences were determined, with GenBank accession numbers.

Strain	Origin	MON/LON a	Kinesin	HASPB amplicon		
				HASPB1	HASPB2	Unpredicted
MHOM/ET/67/HU3 (LV9)	Ethiopia	MON18/LON46	KC342866	KC342849	KC342855	-
MHOM/ET/00/HUSSEN	Ethiopia	MON31/LON42	KC342867	-	-	KC342861
MHOM/ET/72/GEBRE1	Ethiopia	MON82/LON50	-	KC342850	-	-
MHOM/SD/87/UGX-MARROW	Sudan	MON31	KC342868	-	-	KC342862
MHOM/SD/82/GILANI	Sudan	MON30/LON48	KC342869	KC342851	KC342856	-
MHOM/SD/98/LEM3582	Sudan	MON18	KC342870	KC342852	KC342857	-
MHOM/SD/XX/SUDAN1	Sudan	MON18	KC342871	-	KC342858	-
MCAN/SD/98/LEM3556	Sudan	MON82	-	KC342853	KC342859	-
MHOM/SD/97/LEM3458	Sudan	MON18	-	KC342854	KC342860	-
IMAR/KE/62/LRC-L57	Kenya	MON37/LON44	KC342872	-	-	KC342863
MHOM/KE/67/MRC(L)3	Kenya	MON37/LON44	-	-	-	KC342864
MCAN/IQ/81/SUKKAR 2	Iraq	LON43	-	-	-	KC342865

a MON and LON reference numbers refer to multilocus sequence enzyme electrophoresis (MLEE) profiles.

**Table 2:** GenBank sequences used in comparisons.

Strain	Origin	Gene product	GenBank	Reference
MHOM/BR/82/BA-2,C1	Brazil	Kinesin LcKin/rK39	L07879	[7]
MHOM/IN/KE16/1998	India	Kinesin/Ld-rKE16	AY615886	[10]
Morena^a^	India	Kinesin	DQ648599	[11]
MHOM/IN/80/DD8	b	Kinesin/rKRP42	AB256033	[12]
MHOM/SD/62/1S-CL2D	Sudan	Kinesin/LdK39	DQ831678	[13]
MCAN/ES/98/LLM-877 (JPCM5)^c^	Spain	Kinesin	XM_00146426^e^	[18]
MHOM/NP/2003/BPK282/0cl4^d^	Nepal	Kinesin	FR799601^e^	[19]
MHOM/ET/67/L28 (LV9)	Ethiopia	HASPB1	AJ011810	[14]
MHOM/ET/67/L28 (LV9)	Ethiopia	HASPB2	AJ011809	[14]
MHOM/NP/2003/BPK282/0cl4^d^	Nepal	HASPB	FR799601^e^	[19]

a Described in [11] as clinical isolate, not laboratory strain, so WHO code not given here.

b Described in [12] as isolated from a Bangladeshi patient, but MHOM/IN/80/DD8 is also reported as being from India [6].

c *L. infantum* reference genome.

d *L. donovani* reference genome.

e GenBank numbers refer to locations on the entire chromosome 23 sequence, to which both the kinesin and HASPB BLAST searches map.

### Kinesin homologues

**Figure 1:**
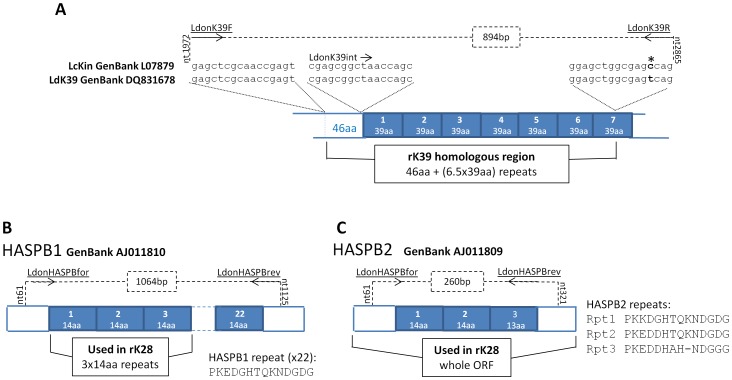
*Leishmania* rK39 and HASPB antigen repeats and the PCR primer target sequences. Repetitive coding regions depicted as filled boxes, PCR primers underlined, and 5′ and 3′ binding positions with amplicons are indicated by dashed lines. [A] Kinesin gene comparison for primer design (* = non-conserved nucleotide). [B] HASPB1 GenBank sequence displays 22× perfect 14aa repeats. [C] HASPB2 GenBank sequence displays 3 imperfect repeats.

PCR primers LdonK39F (gagctcgcaaccgagt) and LdonK39R (ctgrctcgccagctcc) were designed for this study based on a comparison of LcKin and LdK39 coding sequences (Figure 1A), and were targeted to amplify the 894 bp region of the *L. donovani* kinesin gene that is incorporated into the diagnostic antigen rK39. The level of conservation in the sequences encoding the 39-aa repeats predicts that primer LdonK39R would be expected to anneal to multiple sites within the kinesin gene template, generating multiple amplicons. Amplification reactions were performed in a total volume of 20 ul, and comprised of 1× NH_4_ Reaction buffer supplemented with 1.5 mM MgCl_2_ (Bioline, UK), 200 µM dNTPs (New England Biolabs, UK), 10 pmol of each primer, and 1 U BioTaq DNA polymerase (Bioline). Amplification conditions were: 1 cycle of 94°C, 2 mins; 25 cycles of 94°C for 30 secs, 55°C for 30 secs, 72°C for 1 min; 1 cycle of 72°C for 5 mins. PCR products were separated by electrophoresis on 1.5% agarose gels (Bioline). Amplicons corresponding to rK39 homologues were excised and purified from gels using QIAquick Gel Extraction Kit (Qiagen), cloned into pGEM-T easy vector (Promega, UK), and transformed into XL1-blue *E. coli* on blue-white selection. Primers Sp6/T7 and M13for/rev were used to sequence from the vector (between 2–5 colonies per strain were taken forward for sequencing, except UGX-MARROW and SUDAN1, where due to difficulty in cloning the corresponding inserts only one clone was sequenced); in addition, LdonK39int (cgagcggctaaccagc), which binds to the 3′ end of the non-repeat region immediately upstream of the repeats, was used as an internal sequencing primer (Figure 1A). Sequences were analysed using BioEdit [20].

### HASPB

PCR primers LdonHASPBfor (cataaaaccactgaggc) and LdonHASPBrev (atcttcgttcttctcctg) were designed for this study to flank the repeat regions of the HASPB1 ORF, amplifying a 1064 bp product (Figure 1B). Due to the identity between HASPB1 and HASPB2 at the primer binding sites, a 260 bp product would also be predicted to be amplified from HASPB2 by these primers (Figure 1C). Composition of PCR mix was as described above for kinesin, except that 40 µM dNTPs were used. Amplification conditions were: 1 cycle of 94°C, 2 mins; 25 cycles of 94°C for 30 secs, 50°C for 30 secs, 72°C for 1 min; 1 cycle of 72°C for 10 mins. PCR products were separated by electrophoresis on 1% agarose gels (Bioline). Amplicons were excised and purified from gels using QIAquick Gel Extraction Kit, and sequenced directly using the amplification primers. Sequences were analysed using BioEdit.

### Accession numbers

Kinesin nucleotide sequences derived in this manuscript are available under GenBank accession numbers KC342866- KC342872. HASPB Nucleotide sequences derived in this manuscript are available under GenBank accession numbers KC342849- KC342865.

## Results

### East African *L. donovani* kinesin diversity

**Figure 2:**
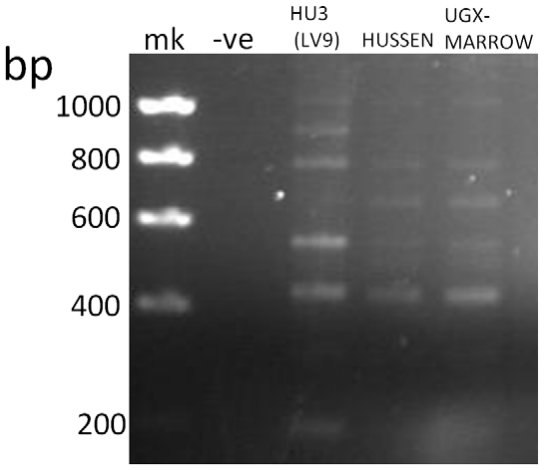
Multiple amplicons corresponding to kinesin tandem repeats are produced by PCR primers LdonK39F and LdonK39R. Amplifications from strains HU3 (LV9), Hussen, and UGX-MARROW, are depicted. Major amplicon sizes differ by 117 bp, the size of the nucleotide sequence encoding the 39aa repeat in the kinesin gene; mk = Hyperladder I (Bioline).

Multiple kinesin amplicons were produced using a combination of primers LdonK39F, which binds to the non-repeat region and LdonK39R, because the latter primer binds to nucleotide sequence that is conserved across the repeats. An example is shown in Figure 2. Sequencing of cloned amplicons containing the rK39 homologous sequences into plasmid vectors revealed the presence of nucleotide and predicted amino acid diversity among East African *L. donovani* strains. Table 3 shows the amino acid polymorphisms that were found among the East African strains, together with their divergence from *L. infantum* (*L. chagasi*) derived LcKin rK39, and alongside the GenBank sequence for *L. donovani* derived kinesin LdK39 used in rK28. Substitutions between a non-charged and a charged residue (D^−^, E^−^, H^+^, K^+^, R^+^) in comparison with the LcKin rK39 sequence are shown underlined.

**Table 3:** rK39 polymorphism among East African *L. donovani* strains and in comparison with the LcKin diagnostic rK39 sequence.

	**P** c	**Rpt1**					**Rpt2**								**Rpt3**							
**Amino Acid**	41	4	21	23	27	39	6	7	10	21	23	24	27	39	7	8	10	21	23	27	35	39
LcKin(used in rK39) a	C	Q	A	A	S	T	R	E	A	A	A	A	M	T	D	S	E	S	T	M	S	T
LdK39(used in rK28) b	S	L	S	T	M	T	R	D	E	A	A	A	S	A	D	S	E	S	T	T	N	T
MHOM/ET/67/HU3(LV9)	S	L	S	T	M	T	R	D	E	A	A	A	S	A	D	^S^_P_	E	S	T	T	N	T
MHOM/ET/00/HUSSEN	S	Q	A	A	S	A	R	E	E	A	A	A	^S^_P_	A	E	S	A	^S^_A_	^T^_A_	^M^_S_	N	T
MHOM/SD/87/UGX-MAR	S	Q	A	A	S	A	R	E	E	A	A	A	S	A	E	S	A	S	T	M	N	T
MHOM/SD/82/GILANI	S	L	^S^_A_	^T^_A_	^M^_S_	T	R	D	E	^S^_A_	^T^_A_	A	^M^_S_	^T^_A_	D	S	E	^A^_S_	^A^_T_	^S^_T_	N	^A^_T_
MHOM/SD/98/LEM3582	S	L	S	T	M	T	R	D	E	A	A	^V^_A_	S	A	D	S	E	S	T	T	N	T
MHOM/SD/XX/SUDAN1	S	L	S	T	M	T	R	D	E	A	A	A	S	A	D	S	E	S	T	T	N	T
IMAR/KE/62/LRC-L57	S	Q	A	A	S	T	L	E	E	S	T	A	M	A	E	S	E	A	A	S	N	T

	**Rpt4**									**Rpt5**										
**Amino Acid**	2	6	7	10	21	23	27	35	39	2	6	7	16	17	18	21	23	27	35	39
LcKin(used in rK39)	E	R	D	E	S	T	M	S	T	E	R	E	A	S	Q	S	T	M	S	T
LdK39(used in rK28)	E	R	D	E	S	T	M	N	A	E	L	E	K	A	E	A	A	S	N	A
MHOM/ET/67/HU3(LV9)	E	R	D	E	S	T	M	N	A	E	L	E	K	A	E	^A^_V_	A	S	N	A
MHOM/ET/00/HUSSEN	^E^_G_	R	E	^E^_A_	A	^A^_T_	^S^_M_	N	T	^G^_E_	R	E	A	S	Q	A	^T^_A_	S	N	A
MHOM/SD/87/UGX-MAR	G	R	E	A	A	A	S	N	T	G	R	E	A	S	Q	A	T	S	N	A
MHOM/SD/82/GILANI	E	R	D	E	S	T	^T^_M_	N	^T^_A_	E	^R^_L_	^D^_E_	^A^_K_	^S^_A_	^Q^_E_	^S^_A_	^T^_A_	^M^_S_	N	A
MHOM/SD/98/LEM3582	E	R	D	E	S	T	M	N	A	E	L	E	K	A	E	A	A	S	N	A
MHOM/SD/XX/SUDAN1	E	R	D	E	-	-	-	-	-	-	-		-	-	-	-	-	-	-	-
IMAR/KE/62/LRC-L57	E	L	E	E	S	T	M	N	A	E	R	E	A	S	Q	A	A	S	N	A

	**Rpt6**									**Rpt7**					
**Amino Acid**	6	7	16	17	18	21	23	27	39	2	7	10	16	17	18
LcKin(used in rK39)	R	D	A	S	Q	A	A	S	A	E	D	E	A	S	Q
LdK39(used in rK28)	R	D	A	S	Q	S	T	T	T	E	D	E	A	S	Q
MHOM/ET/67/HU3(LV9)	R	D	A	S	Q	S	T	T	T	E	D	E	A	S	Q
MHOM/ET/00/HUSSEN	R	E	A	S	Q	^S^_A_	^T^_A_	^M^_S_	^A^_T_	^E^_G_	E	^E^_A_	^A^_K_	^S^_A_	^Q^_E_
MHOM/SD/87/UGX-MAR	R	E	A	S	Q	A	A	S	T	G	E	A	K	A	E
MHOM/SD/82/GILANI	^L^_R_	^E^_D_	^K^_A_	^A^_S_	^E^_Q_	A	^A^_T_	S	A	E	D	E	A	S	Q
MHOM/SD/98/LEM3582	R	D	A	S	Q	S	T	T	T	E	D	E	A	S	Q
MHOM/SD/XX/SUDAN1	-	-	-	-	-	-	-	-	-	-	-	-	-	-	-
IMAR/KE/62/LRC-L57	^H^_R_	E	A	S	Q	A	A	S	A	E	E	E	A	S	Q

a LcKin is derived from *L. infantum* (*L. chagasi*).

b LdK39 is derived from *L. donovani*.

c P = pre-repeat region.

– = not determined here.

When the seven rK39 homologous repeats of all the East African strains were compared with the LcKin rK39 amino acid sequence, residues 2, 6, 10, 16 and 18 were each affected three or four times by substitutions involving charge changes. In contrast, although there were multiple substitutions in the latter half of the East African rK39 tandem repeats, especially affecting residues 21, 23, 27 and 39, none of these substitutions involved charge changes. Two tracts of the rK39 repeat, residues 11 to 15 and 28 to 34 were perfectly conserved between LcKin39 across all the East African strains examined and across all seven rK39 repeats within those strains.

Sequencing of multiple plasmid clones revealed the presence of alternative residues at single sites within single strains. This was notably widespread for the two strains Gilani and Hussen, in the case of Gilani, including several alternatives within the first and second repeats.

### Comparison of *L. donovani* kinesin diversity between East Africa and South Asia

**Figure 3:**
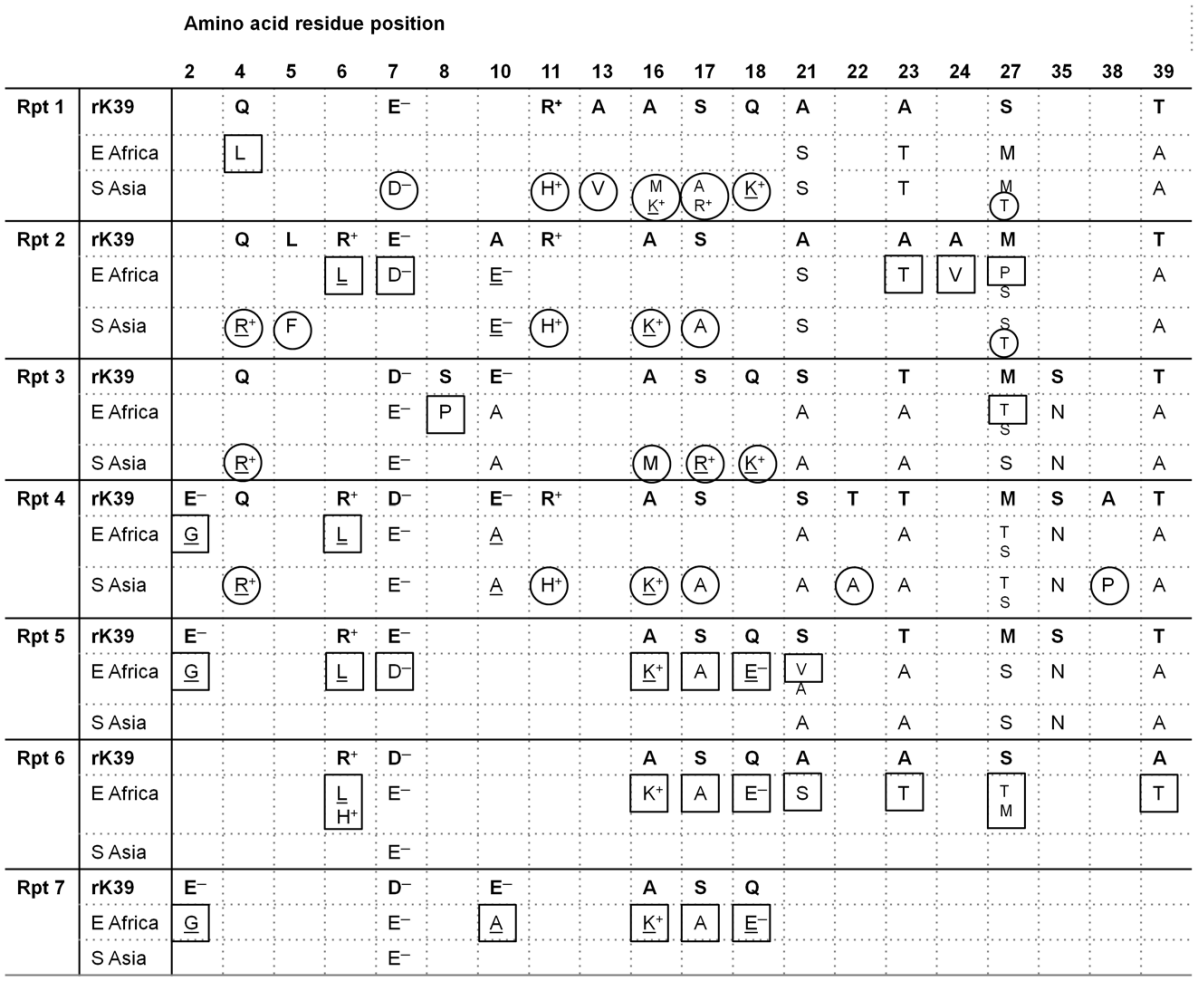
Polymorphisms among seven rK39 repeats of East African and South Asian strains show region-specific substitutions. Changes between charged and non-charged residues are underlined. No residue entered at a particular site indicates conservation of that residue with the corresponding residue of diagnostic rK39. Where two alternative residues are indicated in smaller text these are not necessarily always within the same strain, for clarification compare with Table 3. Region-specific polymorphisms in each repeat are boxed (East Africa) or circled (South Asia).

Figure 3 depicts the composite *L. donovani* kinesin polymorphisms for East Africa and South Asia and the divergence between the two geographic regions, in comparison with the LcKin rK39 derived from *L. infantum* (*L. chagasi*). The East African polymorphisms in Figure 3 incorporate the data obtained here together with the published LdKin (rK28) sequence. Substitutions between non-charged and charged residues (D^−^, E^−^, H^+^, K^+^, R^+^) compared to the LcKin rK39 sequence are shown underlined. East Africa-specific polymorphisms are boxed; South Asia-specific polymorphisms are circled.

In the 46aa non-repeat region the only divergence from *L. infantum* (*L. chagasi*) diagnostic LcKin rK39 was Cys→Ser^41^, found among both South Asian and East African strains (Table 3). Throughout the 6.5×39aa repeats, there were polymorphisms divergent from the diagnostic LcKin rK39 that were unique to each region and other polymorphisms that were common to East Africa and South Asia. For example, Glu→Gly^2^ (Repeats 4, 5, 7) and Arg→His^11^ (Repeats 1, 2, 4) are found only in East Africa and South Asia respectively, whereas in Repeat 1, Ala→Ser^21^, Ala→Thr^23^, Ser→Met^27^, Thr→Ala^39^, which are all conservative changes found in the latter half of the repeats, were found in both regions. Indeed, as for the East African strains (Table 3) all such polymorphisms identified in the latter half of the repeats among South Asian strains are conservative, *i.e.*, a non-charged residue is replaced by another non-charged residue; conversely, non-conservative substitutions between non-charged and charged residues are found in the first half of the repeats. Among the published South Asian sequences charges diverged from those in diagnostic LcKin rK39 at residue 4 (Gln→Arg; Repeats 2, 3, 4) and at residues 16, 17 and 18, for example uncharged to positive at these three sites in repeat 1 (Figure 3).

### Comparison with *L. donovani* complex reference genomes

After the sequencing of the *L. infantum* reference genome [18], Downing et al [19] sequenced the Nepalese *L. donovani* strain BPK282/0cl4 as the reference genome for *L. donovani*. The sequence coding for the non-repeat 46-aa region of rK39 was submitted to NCBI BLAST against the reference genomes. For the *L. infantum* reference genome (Spanish, canine-isolated), there were no differences across the entire rK39 sequence, in accordance with this being the postulated source of *L. infantum* (*L. chagasi*) in South America [3]. For the genome sequence of BPK282, due to the repeat nature of the downstream region, only sequences of the first two repeats could be unequivocally aligned and these were incorporated into Figure 3 for comparisons with the individual repeat sequences generated here. From the unequivocal BLAST alignment information, BPK282 repeat 1 is the same as for rK39 except Thr→Ala^39^; in BPK repeat 2, where only the first 6 residues are unambiguously assembled, Gln→Arg^4^ is present.

### *L. donovani* HASPB

**Figure 4:**
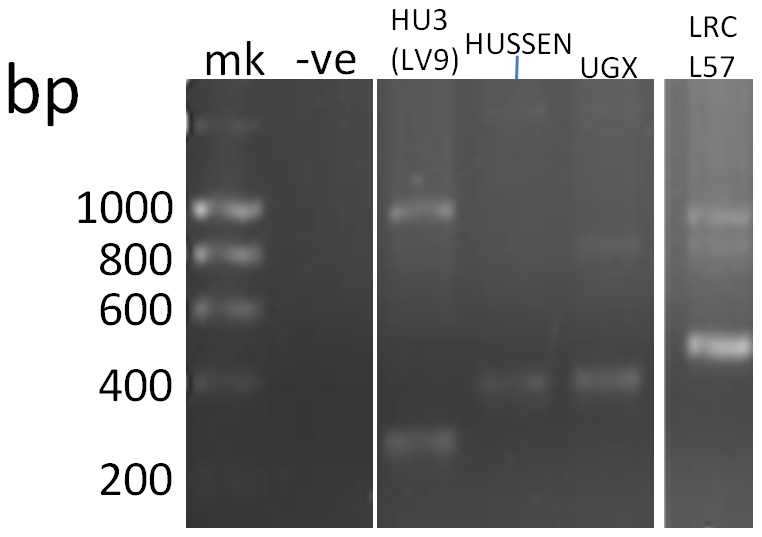
Predicted (1064 bp, 260 bp) amplicons and unpredicted (∼400–500 bp) amplicons with HASPB PCR primers LdonHASPBfor and LdonHASPBrev. Amplifications from strains HU3 (LV9), Hussen, UGX-MARROW, and LRC-L57, are depicted; mk = Hyperladder I.

PCR primers LdonHASPBfor and LdonHASPBrev were designed to bind unique sequences flanking the 22×14aa-repeat coding region of HASPB1 to produce a 1064 bp amplicon. However, with some of the strains studied here in addition to the 1064 bp product the smaller 260 bp amplicon corresponding to HASPB2 (Figure 1C) could also be seen: an example is shown for HU3 (LV9) in Figure 4. However, these primers unexpectedly gave amplicons of ∼400–500 bp for some of the strains (Hussen, UGX-marrow, LRC-L57, MRC(L)3, SUKKAR 2) (Figure 4).

### Comparisons with rK28 HASPB sequences

Strains HU3 (LV9), Gebre1, Gilani, LEM3582, LEM3556, and LEM3458, which gave 1064 bp-amplicons, had identical sequences in their first 3×14aa repeats as the HASPB1 used in diagnostic rK28. However, subsequent repeats, not incorporated into rK28, were imperfect with polymorphic residues at certain sites, such as His→Arg^6^ (repeats 15, 18, 22) and Gly→Ala^14^ (repeats 5, 14, 17) for strains LEM3582, LEM3556, and LV9.

For those strains producing the predicted 260 bp amplicon of the HASPB2 homologue, (Figure 1) a comparison with the rK28 sequence identified a Gly residue inserted between Ala^32^ and Val^33^ preceding the imperfectly repeated 14/13aa region and a Pro→Gln^96^ substitution present after the 14/13aa region. The only polymorphism within the imperfectly repeated 14/13aa region, compared to rK28, was Lys→Glu^49^, at the third residue of first 14aa region, with a consequent charge change.

Interestingly, the strains producing unexpected ∼400–500 bp amplicons (Hussen, UGX-marrow, LRC-57, SUKKAR 2, MRC(L)3) revealed on sequence analysis a HASPB1/HASPB2-like combination composition across their repeats, due to the presence of the central motifs that were HASPB1-like (HTQKN) or HASPB2-like (HAH-N). The HTQKN and HAH-N motifs were not consistently present in the same repeat numbers across these five strains and the HASPB1-like and HASPB2-like sections of the tandem repeat were not necessarily aggregated (Table 4). In the case of LRC/L57, both the 1064 bp (predicted HASPB1-homologue) and ∼500 bp amplicons contained the HASPB2-like motif HAH-N. The Lys→Glu^49^ substitution at the third residue of the first 14aa region, described above, was also present in those strains giving the ∼400–500 bp amplicons.

**Table 4:** Repeat sequences of unexpected amplicons.

	HUSSEN	UGX-Marrow	LRC-L57	MRC(L)3	SUKKAR 2
Rpt 1	LKEDGHTQKNDGDG	LKEDGHTQKNDGDG	PKEDGHTQKNDGDG	PKEDGHTQKNDGGG	LKEDGHTQKNDGDG
Rpt 2	PKEDGHTQKNDGGA	PKEDGHTQKNDGGA	PKEDDHAH-NDGGG	PKEDDHAH-NDGGG	PKEDGHTQKNDGDG
Rpt 3	PKEDGHTQKNDGDG	PKEDGHTQKNDGDG	PKEDDHAH-NDGGG	PKEDDHAH-NDGGG	PKEDDHAH-NDGDG
Rpt 4	PKEDDHAH-NDGDG	PKEDDHAH-NDGDG	PKEDGHTQKNDGDG	PKEDGHTQKNDGDG	PKEDDHAH-NDGDG
Rpt 5	PKEDDHAH-NDGGG	PKEDDHAH-NDGGG	PKEDDHAH-NDGGG	PKEDDHAH-NDGGG	PKEDDHAH-NDGGG
Rpt 6	PKEDDHAH-NDGGA	PKEDDHAH-NDGGG	PKEDDHAH-NDGGG	PKEDDHAH-NDGGG	PKEDDHAH-NDGGG
Rpt 7			PKEDDHAH-NDGGG	PKEDGHTQKNDGGV	
Rpt 8			PKEDGHTQKNDGGV		

Central motifs:

HTQKN = HASPB1-like.

HAH-N = HASPB2 like.

### Comparison with *L. donovani* reference genome

The sequence coding for the pre-repeat region of HASPB1 (not used in rK28, Figure 1A) was submitted to NCBI BLAST against the Nepalese *L. donovani* reference genome, to facilitate correct alignment. Due to the repeat nature of the downstream region, only the first two repeats of the reference genome could be unequivocally aligned for comparisons with the individual sequences generated here. The first two repeats of the reference genome showed the combination composition of central motifs HTQKN in repeat 1 (HASPB1-like) and HAH-N in repeat 2 (HASPB2-like). Thus, the South Asian reference genome has a HASPB1/2 combination structure like the four East African strains Hussen, UGX-marrow, LRC-57, MRC(L)3, and the Iraqi SUKKAR 2, and with the same organisation of repeats 1 and 2 seen in the Kenyan strains LRC-57 and MRC(L)3.

## Discussion

For VL there is a particular need for rapid diagnostic tests (RDTs) that can be unequivocally applied in endemic regions at primary care level and with only basic technical training. Of the available options for serological diagnosis of VL only the immunochromatographic rK39 assay can be considered a point of care test for field application. The IFAT requires a fluorescence microscope. Use of the DAT is constrained by the readout delay, the relative sophistication of the procedure and limited access to lyophilised antigen. In agreement with other studies, Boelaert et al [8] recommend the use of rK39 in South Asia, but report less satisfactory results from East Africa. One factor influencing regional differences in diagnostic performance may be divergence in kinesin gene homologues in East African *L. donovani* from the *L. infantum* (*L. chagasi*)-derived LcKin rK39 sequence. In an attempt to overcome the geographical limitations of the rK39 test a modified recombinant antigen, rK28, has been devised, incorporating segments of the HASPB antigen.

Analyses of *L. donovani* complex isolates with multiple molecular markers have revealed extensive genetic diversity with at least six distinct lineages, and both inter- and intra-lineage diversity. Furthermore, genotyping of isolates from endemic regions has shown an association between genotype and geographical origin [21]–[22]. Kuhls et al [22] used multilocus microsatellite typing (MLMT) for high resolution comparison of the six global *L. donovani* genetic lineages, and showed that allelic diversity in Africa was greater than in India; a subsequent MLMT study also described comparative homogeneity among South Asian *L. donovani* [23]. Thus, the relative homogeneity of South Asian *L. donovani* and greater diversity in East Africa, if mirrored in diversity of the kinesin gene, is one potential explanation of the more limited efficacy of diagnostic rK39 in East Africa.

Here we determined across a panel of East African strains the diversity of the kinesin repeat region homologous to that used in both the rK39 diagnostic antigen (repeats 1 to 6.5) and the rK28 recombinant antigen (repeats 1 and 2). We then compared this diversity to that among predetermined South Asian sequences.

Surprisingly, in view of the proven value of the rK39 RDT in South Asia, there is considerable amino acid diversity among kinesin repeats 1 to 7 of South Asian strains as well as those from East Africa (Figure 3). Alternative residues at multiple sites suggested that Gilani and Hussen are heterozygous, although sequences were not obtained from DNA clones and therefore multiclonality rather than heterozygosity cannot be excluded. Across all strains the diversity was particularly notable in the first half of each rK39 repeat, because it involved charge changes at residues 2, 6, 10, 16, and 18, with only a short stretch of consistently conserved residues (11 to 15). Such charge changes among the South Asian strains are very likely to disrupt antigenic epitopes, if present. As far as we are aware epitopes within or across adjacent rK39 repeats have not yet been precisely mapped. However, we would predict that the diagnostic epitopes lie in the latter half of the rK39 repeat, where, although there is extensive diversity, the changes are conservative, none involving charge, and there is one stretch of residues (28 to 34) that is entirely conserved across all isolates and across all seven repeats. This is consistent with the recent work of Costa et al [24] in which antigen prediction software led to the synthesis of a 22mer peptide (ESTTAAKMSAEQDRESTRATLE) encompassing the region of rK39 from residue 20 of repeat 3 to residue of 2 of repeat 4, a sequence which is also found in the next two repeats. In that study, the peptide was recognised in ELISA by Brazilian sera from symptomatic and asymptomatic canine VL and symptomatic human VL. However, our study shows that at least 5 of the residues within that 22mer are polymorphic in East Africa.

Kinesin repeat divergence from the rK39 diagnostic recombinant is complex, and some divergence is common to both South Asia and East Africa, for example at residues 7, 10, 21, 23 (Figure 3). Nevertheless, there were polymorphisms unique to each region, for example at residues 4, 16, and 18, consistent with the expectation that differential sensitivities of rK39 assays may be partially attributable to positional and physiochemical polymorphisms of the kinesin gene. The identification and confirmation of region-specific polymorphisms may be limited by the amount of sequence data so far available. Also diversity beyond the seven sequenced repeats of the kinesin tandem array may present a wider range of epitopes for immunological recognition.

The differential immunocompetence and antibody titres of East African and South Asian human populations in the context of VL remains to be thoroughly explored, as a major alternative or contributing explanation of variable rK39 test sensitivities. Interestingly a recent WHO/TDR evaluation [9] of a rapid diagnostic test based on rKE16 antigen, a LcKin homologue derived from an Indian *L. donovani* [10], reported markedly lower sensitivities against Brazilian and East African sera, compared to South Asian. The same evaluation [9] also reported a lower sensitivity of rK39-based tests with Brazilian sera. However, the rK39 antigen is derived from a Brazilian *L. infantum* (*L. chagasi*) strain [7], which originated from Iberia in Europe and is considered to be relatively homogeneous, based on microsatellite typing [3]. Thus, kinesin diversity among Brazilian *L. infantum* should be explored further and comparative immune response levels among Brazilian and South American populations.

In the rK28 recombinant diagnostic antigen, designed to improve on performance of the rK39 test, highly conserved HASPB repetitive amino acid sequences flank the first two repeats of Ldk39, derived from an East African strain. Alce et al [14], following the work of McKean et al [25], amplified two homologues of the *L. major* Gene B protein from *L. donovani*, naming the ORF of the larger HASPB1 and that of the smaller HASPB2. Recombinant HASPB1 has been used in animal vaccine models [26]–[27], and HASPB has a role in *L. major* differentiation within the sandfly vector *P. papatasi* [28]. The PCR primers used in the current study, like those used by Alce et al [14], anneal to regions flanking HASPB repeat regions and generate two PCR products of predicted size corresponding to HASPB1 and HASPB2, compatible with the presence of two distinct HASPB1 and HASPB2 loci in the genome, as concluded for the *L. major* genome [29]. However, several strains produced unexpected sized amplicons with a mix of HASPB1 and HASPB2 motifs within these PCR products, indicating a structural reorganisation of HASPB in these strains. Similarly, Haralambous et al [30] in K26, a homologue of HASPB, also found unexpected amplicons for the Hussen strain as compared to Gebre1 and Gilani. Nevertheless, despite this structural reorganisation, there was limited HASPB amino acid diversity across the strains. There was only one polymorphism compared to rK28 that involved a charge change, Lys→Glu^49^ in HASPB2, potentially affecting antigenicity. A recent study [31] reports HASPB repeat sequence diversity in Indian *L. donovani* strains; a comparison with sequences derived here shows that among those East African strains producing unexpected sized amplicons (Table 4), there is the presence of repeat sequence types that are also predominant among Indian strains (PKEDDHAHNDGGG, PKEDGHTQKNDGDG, PKEDDHAHNDGDG). In addition we find repeat types not reported from Indian strains, for example repeats beginning with Lys in place of Pro (Hussen, UGX-Marrow, SUKKAR 2), or ending in Val (LRC-L57, MRC(L)3).

We did not have patient samples from which to attempt direct amplification of kinesin and HASPB and confirm diversity *in situ*. Nevertheless the polymorphisms described here do not occur stochastically across strains but at sites of reported kinesin diversity, or in the case of the HASPB2, are consistent among strains (Ala^32^-Gly-Val^33^ or Pro→Gln^96^). Furthermore, the strains were cryopreserved, not subjected to prolonged passage *in vitro*, and sequences were determined bi-directionally and/or repeated to confirm their validity.

We have undertaken the most comprehensive analysis of diagnostic kinesin and HASPB antigen diversity in East African strains to date. We show that there is extensive kinesin genetic diversity among strains and between East Africa and South Asia, with ample scope for influencing performance of rK39 diagnostic assays. Future research should both widen and focus the genomic comparisons between strains and also compare immune competence profiles among East African, Brazilian and South Asian populations as an alternative or contributory factor to variable RDT performance. There are broader implications from these findings. Firstly, we see the crucial importance of sustaining accessible collections of *Leishmania* strains representative of genetic lineages and global diversity. Secondly, due to present limitations in the capacity of whole genome sequencing to assemble complex antigen gene families, especially those comprised of repeated sequences, targeted analysis of individual strains is also necessary for these comparisons of genetic diversity. Thirdly, it is evident that comparative genomics has a vital role in guiding the optimisation of recombinant or synthetic diagnostic antigens. Not only are even better diagnostic tests needed for diagnosis of VL but biomarkers are urgently required to distinguish symptomatic cases, asymptomatic infections at risk or not of progression to VL, and post-treatment outcome (relapse *versus* cure).
